# Prognostic value of baseline QFR in single-vessel intermediate coronary stenosis

**DOI:** 10.3389/fcvm.2025.1726729

**Published:** 2026-01-09

**Authors:** Yanfeng Lu, Abdulrahman AlQazzaz, Jasmine Yimeng Bao, Gary S. Mintz, Jiahao Feng, Yong Zhang, Shanshan Gao, Qiang Song, Feifei Ning, Xin Huang, Ning Guo

**Affiliations:** 1 Department of Cardiovascular Medicine, The First Affiliated Hospital of Xi’an Jiaotong University, Xi’an, Shaanxi, China; 2 Sidney Kimmel Medical College, Thomas Jefferson University, Philadelphia, PA, United States; 3 Cardiovascular Research Foundation, New York, NY, United States

**Keywords:** coronary artery disease, grey-zone, percutaneous coronary intervention, prognosis, quantitative flow ratio

## Abstract

**Background and objectives:**

The prognostic value of baseline Quantitative Flow Ratio (QFR) in real-world patients remains unclear. This study aimed to evaluate the prognostic value of baseline QFR and its three-tier model—low, grey-zone, and high QFR—in patients with single-vessel intermediate stenosis.

**Methods:**

This retrospective study included 478 patients with QFR between 0.70 and 0.90 who underwent coronary angiography between May and June 2023. Patients were stratified into Low (0.70–0.74), Grey-Zone (0.75–0.85), and High (0.86–0.90) QFR groups. The primary endpoint was major adverse cardiac events (MACE); the key secondary endpoint was target vessel failure (TVF). Kaplan–Meier and Cox proportional hazards models were used to evaluate outcomes.

**Results:**

During the 18-month follow-up period, MACE incidence was 13.5%, 6.6%, and 3.4% in Low, Grey-Zone, and High QFR groups, respectively (*P* = 0.008), mainly driven by MI (3.2% vs. 1.4% vs. 0.0%, *P* = 0.029) and ischemia-driven revascularization (11.5% vs. 6.1% vs. 2.3%, *P* = 0.004), including ID-TVR (6.7% vs. 3.3% vs. 1.1%, *P* = 0.027). Each 0.01 increase in QFR was associated with a 7.23% lower risk of MACE (*P* = 0.012) and a 10.04% lower risk of ID-TVR (*P* = 0.011). Multivariate analysis confirmed QFR group as an independent predictor, with a per-category decrease from High to Low QFR associated with an 86% higher risk of MACE (adjusted HR = 1.858, 95% CI 1.038–3.326, *P* = 0.037) and a 2.33-fold higher risk of ID-TVR (adjusted HR = 2.333, 95% CI 1.004–5.510, *P* = 0.049).

**Conclusions:**

Baseline QFR and its three-tier stratification across the grey-zone and adjacent ranges show a continuous association with adverse events in single-vessel intermediate coronary stenosis, supporting its role in functional evaluation, risk stratification, and prognostic prediction.

## 1 Introduction

Intermediate coronary stenosis present a diagnostic and therapeutic dilemma in patients with coronary artery disease (CAD). Inaccurate determination of ischemic relevance may lead to unnecessary percutaneous coronary intervention (PCI) or undertreatment. Fractional flow reserve (FFR)—invasive measurement of pressure gradient under hyperemic conditions—has proven its prognostic value and demonstrated reduction in inappropriate interventions (1–6). Yet, FFR adoption remains limited due to procedural complexity, cost, and need for pharmacologic vasodilators.

Quantitative Flow Ratio (QFR) has emerged as a novel, non-interventional technique that computes an FFR-equivalent value from one or two angiographic projections without pressure wires or hyperemic agents (7, 8). Studies (7–11) have demonstrated high diagnostic accuracy for QFR when compared with FFR. Additionally, prospective trials (12, 13) suggest QFR-guided treatment can reduce major adverse cardiac events (MACE) relative to angiography-guided strategies. However, these earlier studies predominantly focused on dichotomous QFR thresholds (often 0.80) and did not explore the relationship between the full continuum of baseline QFR values and prognosis.

The present study evaluated the prognostic value of baseline QFR, both as a continuous variable and in three distinct strata (Low: 0.70–0.74; Grey-Zone: 0.75–0.85; High: 0.86–0.90) for single-vessel intermediate stenosis.

## 2 Materials and methods

### 2.1 Study design and population

**Figure 1:**
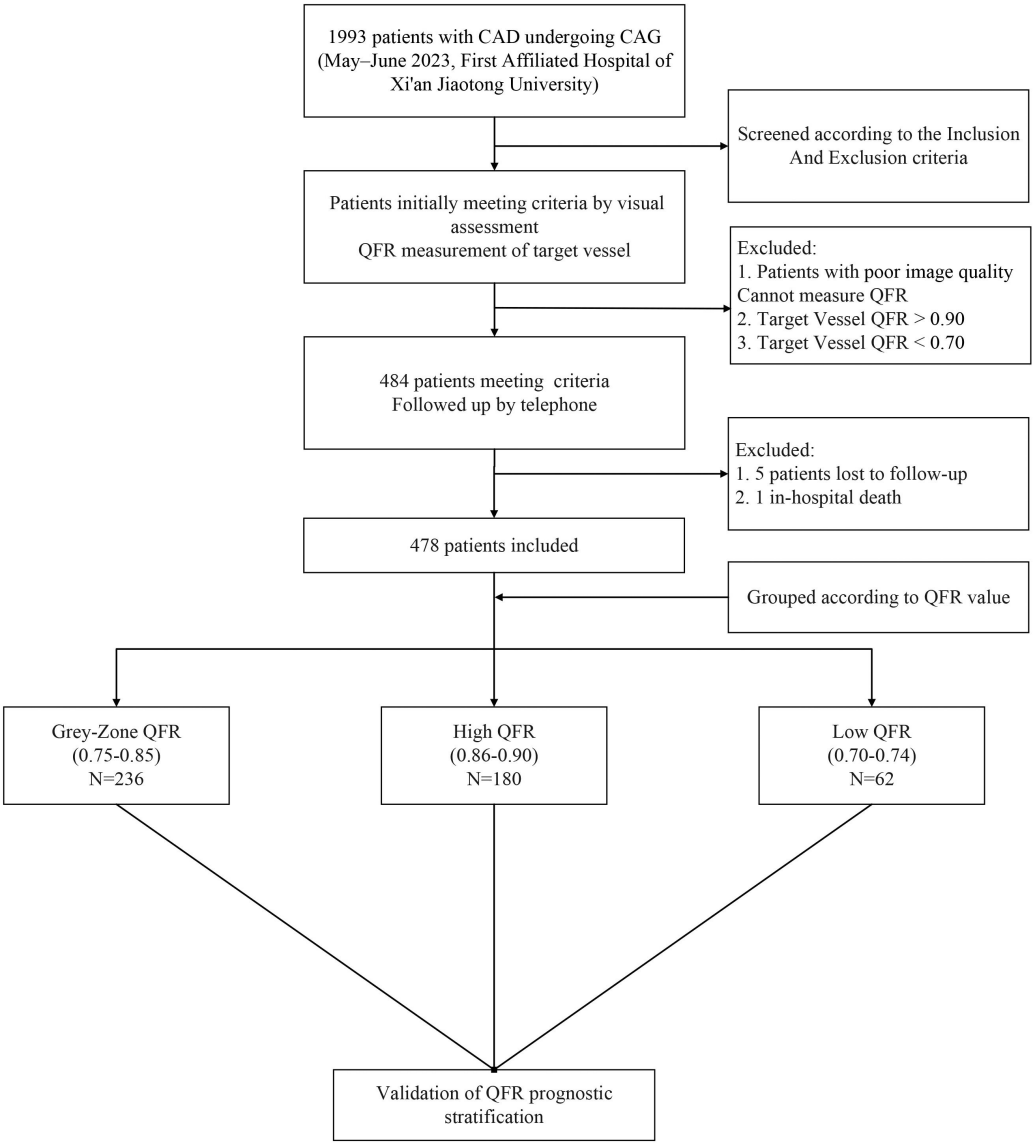
Enrollment flow chart.

This was a single-center, retrospective cohort study that included CAD patients undergoing coronary angiography at the First Affiliated Hospital of Xi'an Jiaotong University (Xi'an, China) between May and June 2023. Inclusion criteria were angiographic evidence of a single coronary artery with intermediate stenosis (diameter stenosis 30%–70% by visual assessment) and retrospective QFR measurements between 0.70 and 0.90. Other coronary arteries were either free of significant disease or had undergone PCI for severe stenoses. Exclusion criteria included poor image quality, hemodynamic instability, >1 coronary artery with intermediate stenosis, left main or chronic total occlusion lesions, ostial lesions, stented target vessel, culprit lesions, in-hospital death, incomplete clinical data, or lost to/refusal of follow-up. The study flowchart is shown in Figure 1.

The studies involving humans were approved by the Clinical Research Ethics Committee of the First Affiliated Hospital, School of Medicine(SOM), Xi’an Jiaotong University. The studies were conducted in accordance with the local legislation and institutional requirements. The ethics committee/institutional review board waived the requirement of written informed consent for participation from the participants or the participants' legal guardians/next of kin because the retrospective nature of the study, using of de-identified patient records and posed no additional risk to participants.

### 2.2 Procedures and QFR measurement

Coronary angiography and PCI were performed following standards of practices. μQFR (referred to as QFR in this study) was independently measured by two trained researchers following standardized procedures (8), and the mean value was recorded. In general, angiographic images were imported from the PACS system into the software (Angio Plus, Shanghai Pulsed Medical Technology Co., Ltd., Shanghai, China), which automatically extracted main coronary artery and side branch lumen contours with calculations based on Murray's law. For eccentric lesions, a second angiographic projection was selected for calibration. Selection criteria prioritized minimal vessel overlap or foreshortening, clear visualization of the lesion lumen, adequate contrast filling, and complete dynamic sequence of vessel opacification. The software generated a structured report, including lesion length, minimum lumen diameter (MLD), and diameter stenosis (DS).

The SYNTAX Score I was independently calculated by trained interventional researchers using the online tool (https://syntaxscore.org). Lesions were categorized as low-risk (≤22), intermediate-risk (23–32), or high-risk (≥33) (14).

### 2.3 QFR stratification

Based on the definition of the QFR grey-zone adopted in FAVOR III China (12, 15), vessels with QFR values between 0.75 and 0.85 were defined as the grey-zone. According to the measured QFR of the target vessel, patients were classified into three groups: Low QFR group (0.70 ≤ QFR < 0.75, hereafter referred to as Low), Grey-zone QFR group (0.75 ≤ QFR ≤ 0.85, hereafter referred to as Grey-zone), and High QFR group (0.85 < QFR ≤ 0.90, hereafter referred to as High).

### 2.4 Data collection and endpoints

Baseline demographics, clinical history, angiographic characteristics, and treatment modalities (PCI vs. medication) were collected. The primary endpoint was major adverse cardiovascular events (MACE), defined as a composite of cardiac death, non-fatal myocardial infarction (MI), and ischemia-driven revascularization (IDR). The key secondary endpoint was target vessel failure (TVF), including cardiac death, target vessel MI (TV-MI), and target vessel ischemia-driven target vessel revascularization (ID-TVR). Other Secondary endpoints included the individual components of MACE and TVF, as well as all-cause mortality. Unless an unequivocal non-cardiac cause was identified, any death of unknown cause was classified as cardiac death. Follow-up was conducted primarily by telephone, supplemented by review of electronic medical records when necessary. Investigators responsible for follow-up were blinded to QFR results. All clinical events were independently adjudicated by two experienced cardiologists blinded to QFR results; discrepancies were resolved by consensus or, if necessary, by consultation with a third senior cardiologist.

### 2.5 Statistical analysis

All data were entered using SPSS version 27.0, and statistical analyses were performed using SPSS 27.0 or R version 4.4.2. Continuous variables were presented as mean ± standard deviation (SD) or median with interquartile range (IQR: P25, P75), depending on their distribution. Categorical variables were expressed as counts and percentages and were compared using the chi-square test or Fisher's exact test, based on the expected frequencies. For ordinal categorical variables, the linear-by-linear association test was used to assess trends. Clinical event data were analyzed using the Kaplan–Meier Analysis to estimate cumulative incidence, expressed as the number of events and corresponding cumulative event rates. Survival curves were compared using the log-rank test. Restricted Cubic Splines (RCS) are used to analyze the relationship between continuous variables and outcomes, employing nonlinear tests to determine linearity. Cox proportional hazards regression models were constructed to estimate hazard ratios (HRs) and 95% confidence intervals (CIs), identify independent risk factors, and adjust for confounding variables. Subgroup analyses and interaction testing were also performed. Model coefficients (β) were tested using the Wald test.

Unless otherwise stated, all tests were two-sided, and a *P* value < 0.05 was considered statistically significant.

## 3 Results

### 3.1 Baseline characteristics

A total of 1,993 patients undergoing coronary angiography were screened. After visual assessment, 484 patients were preliminarily eligible with target vessel QFR values between 0.70 and 0.90. Five patients were lost to follow-up and one patient died in-hospital. 478 patients were finally included in the study: 62 (12.97%) in the low QFR group, 236 (49.4%) in the grey zone group, and 180 (37.7%) in the high QFR group. Table 1 presents the baseline clinical characteristics of the enrolled patients, stratified by target vessel QFR values. There were no significant differences in age, sex, BMI, diabetes mellitus, hypertension, smoking history, history of myocardial infarction, or statin use among the groups. Laboratory findings were also similar among groups. However, the proportion of patients diagnosed with hyperlipidemia differed significantly (*P* = 0.021), with the grey-zone QFR group showing the highest proportion (44.5%). Also, the low QFR group had a higher proportion of prior PCI (41.9%, *P* = 0.025).

**Table 1:** Baseline clinical characteristics stratified by QFR group.

Clinical characteristics	Low	Grey-Zone	High	*P* value
	(*N* = 62)	(*N* = 236)	(*N* = 180)	
Age, years	64.5 (56.8, 71.0)	64.0 (57.0, 70.0)	64.0 (54.2, 70.8)	0.620
<65	31 (50.0)	124 (52.5)	94 (52.2)	0.937
≥65	31 (50.0)	112 (47.5)	86 (47.8)	
Sex (*n*, %)				0.247
Male	46 (74.2)	177 (75.0)	122 (67.8)	
Female	16 (25.8)	59 (25.0)	58 (32.2)	
BMI, kg/m^2^	24.4 ± 2.9	24.7 ± 3.3	24.7 ± 3.3	0.789
<30	60 (96.8)	219 (92.8)	170 (94.4)	0.473
≥30	2 (5.6)	17 (7.2)	10 (5.6)	
Diabetes mellitus (*n*, %)	28 (45.2)	106 (44.9)	66 (36.7)	0.204
Hypertension (*n*, %)	39 (62.9)	153 (64.8)	103 (57.2)	0.280
Hyperlipidemia (*n*, %)	17 (27.4)	105 (44.5)	63 (35.5)	0.021
History of Statins (*n*, %)	41 (66.1)	134 (56.8)	91 (50.6)	0.092
Smoking (*n*, %)				0.451
Never	23 (37.1)	86 (36.4)	79 (43.9)	
Former	11 (17.7)	39 (16.5)	19 (10.6)	
Active	28 (45.5)	111 (47.0)	82 (45.6)	
Prior MI (*n*, %)	15 (24.2)	41 (17.4)	26 (14.4)	0.212
Prior PCI (*n*, %)	26 (41.9)	59 (25.0)	47 (26.1)	0.025
LDL-C, mmol/L	1.71 (1.26, 2.25)	1.96 (1.50, 2.67)	1.91 (1.44, 2.59)	0.081
HbA1c, %	6.3 (5.9, 6.8)	6.3 (5.8, 7.0)	6.1 (5.8, 6.8)	0.229
eGFR, mL/min/1.73 m^2^				0.799
≥60	58 (93.5)	223 (94.5)	172 (95.6)	
<60	4 (6.5)	13 (5.5)	8 (4.4)	
LVEF (*n*, %)				0.258
>45	51 (82.3)	209 (88.6)	162 (90.0)	
≤45	11 (17.7)	27 (11.4)	18 (10.0)	

Angiographic characteristics by QFR Group have been summarized in Table 2. There were significant differences in angiographic parameters, including lesion length, MLD, and DS (all *P* < 0.001). Patients in the high QFR group generally exhibited less complex coronary lesions, less severe calcification (*P* < 0.001), fewer tandem lesions (*P* = 0.025), and fewer diseased vessels (*P* = 0.003). The SYNTAX Score in High QFR Group was also lower (*P* < 0.001). As a result, patients in the high QFR group were least likely to undergo PCI (*P* < 0.001).

**Table 2:** Angiographic characteristics stratified by QFR group.

Angiographic characteristics	Low	Grey-zone	High	*P* value
	(*N* = 62)	(*N* = 236)	(*N* = 180)	
Length, mm	34.26 (23.00, 43.51)	28.21 (18.70, 38.23)	20.30 (12.97, 31.08)	<0.001
MLD, mm	1.37 (1.18, 1.67)	1.58 (1.30, 1.99)	1.84 (1.56, 2.20)	<0.001
DS, %	53.0 (47.5, 57.8)	47.6 (42.4, 52.6)	40.9 (36.8, 44.9)	<0.001
QFR	0.72 (0.70–0.73)	0.82 (0.79, 0.84)	0.89 (0.88, 0.90)	<0.001
No. of diseased vessels				0.003
1 (*n*, %)	7 (11.3)	38 (16.1)	41 (22.8)	
2 (*n*, %)	29 (46.8)	60 (25.4)	64 (35.6)	
3 (*n*, %)	26 (41.9)	138 (58.5)	75 (41.7)	
Calcification (*n*, %)	30 (48.4)	96 (40.7)	44 (24.4)	<0.001
Tandem lesion (*n*, %)	20 (32.3)	49 (20.8)	29 (16.1)	0.025
SYNTAX Score Ⅰ	25.0 (17.0, 36.5)	26.0 (16.2, 36.8)	19.0 (10.5, 29.5)	<0.001
Low (≤22)	25 (40.3)	98 (41.5)	109 (60.6)	<0.001
Intermediate (23–32)	19 (30.6)	59 (25.0)	39 (21.7)	
High (≥33)	18 (29.0)	79 (33.5)	32 (17.8)	
Treatment (*n*, %)				<0.001
Medication	27 (43.5)	159 (67.4)	142 (78.9)	
PCI	35 (56.5)	77 (32.6)	38 (21.1)	

### 3.2 Prognostic value of QFR

**Figure 2:**
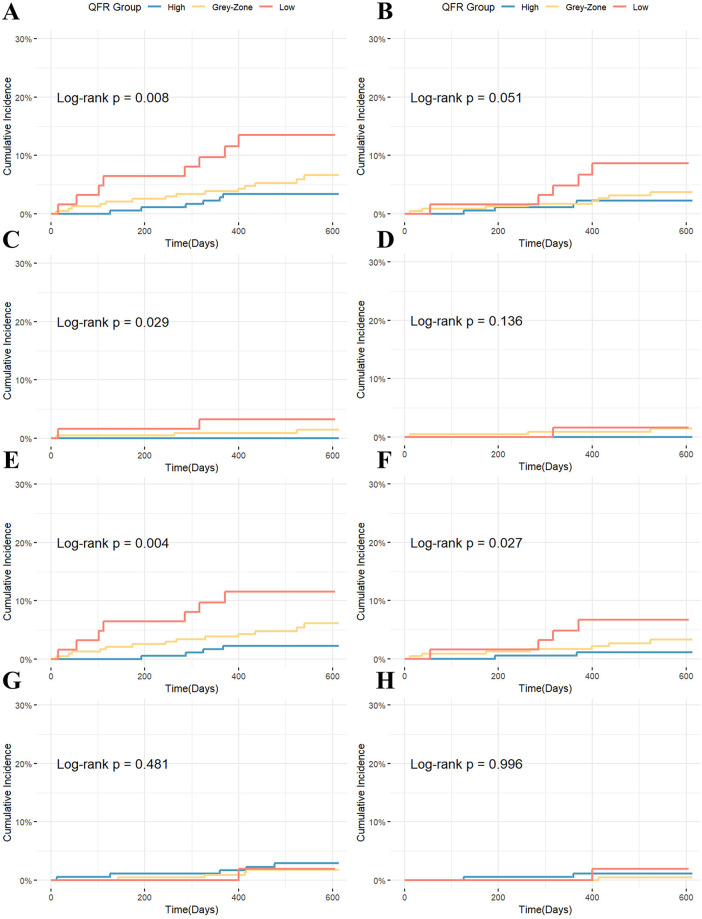
Kaplan–Meier curves for clinical endpoints stratified by QFR group: **(A)** MACE; **(B)** TVF; **(C)** MI; **(D)** TV-MI; **(E)** IDR; **(F)** ID-TVR; **(G)** All-cause mortality; **(H)** Cardiac death.

During the median 18-month follow-up, a total of 28 patients experienced MACE. From high to low QFR groups, the cumulative incidence of MACE increased stepwise (log-rank *P* = 0.008). For the key secondary outcome (TVF), the cumulative incidence showed a similar increasing trend from high to low QFR groups (*P* = 0.051). The difference in MACE was mainly driven by higher rates of IDR (*P* = 0.004) and myocardial infarction (*P* = 0.029) in the low QFR group. The difference in TVF was mainly driven by different rates of ID-TVR rates among groups (*P* = 0.027). Other secondary outcomes (TV-MI, cardiac death, all-cause mortality) showed no significant differences (Table 3, Figure 2). After stratification by treatment strategy (PCI or medical therapy), the association between QFR group and events remained statistically significant, especially in Medication Group (Supplementary Table S1).

**Table 3:** Clinical outcomes stratified by QFR group.

Clinical Outcomes	Low	Grey-Zone	High	*P* value
	(*N* = 62)	(*N* = 236)	(*N* = 180)	
Follow-up, days	558 (488, 578)	552 (523, 578)	555 (503, 579)	0.198
All-cause mortality	1 (1.9)	4 (1.8)	5 (2.9)	0.481
MACE	8 (13.5)	14 (6.6)	6 (3.4)	0.008
Cardiac death	1 (1.9)	1 (0.5)	2 (1.1)	0.996
MI	2 (3.2)	3 (1.4)	0 (0)	0.029
IDR	7 (11.5)	13 (6.1)	4 (2.3)	0.004
TVF	5 (8.6)	8 (3.7)	4 (2.2)	0.051
Cardiac death	1 (1.9)	1 (0.5)	2 (1.1)	0.996
TV-MI	1 (1.6)	3 (1.4)	0 (0)	0.136
ID-TVR	4 (6.7)	7 (3.3)	2 (1.1)	0.027

RCS analysis (Supplementary Figure S1) demonstrated a significant association between QFR and MACE (*P* = 0.026), with no apparent nonlinear trend observed (*P* for nonlinear = 0.538). In the univariable Cox proportional hazards analysis (Supplementary Table S2), QFR as a continuous variable was inversely associated with MACE (*P* = 0.012). Each 0.01-unit increase in QFR was associated with a 7.23% reduction in MACE risk, and the risk increased stepwise across QFR Groups from High to Low (HR = 2.067, 95% CI 1.200–3.558, *P* = 0.009). Among other potential predictors, albumin (ALB), eGFR, and SYNTAX risk group were also significantly associated with MACE. The proportional hazards assumption was tested and satisfied for all variables, as indicated by Schoenfeld residuals (*P* > 0.05).

Variables with a *P* < 0.10 in the univariable analysis were entered into the model using the forward likelihood ratio (LR) method. Although treatment strategy was not statistically significant in the univariable Cox analysis (*P* = 0.362), it was forcibly retained in the model. No multicollinearity was detected among the included variables, based on collinearity diagnostics (0 < VIF < 5 and Tolerance > 0.1).

In the final multivariable Cox model (Table 4), QFR group, SYNTAX risk group, eGFR, and treatment strategy were retained. After adjusting for eGFR, SYNTAX score, and treatment, the predictive effect of the QFR group on MACE remained statistically significant, with a per-category decrease from the High QFR group to the Low QFR group associated with an 86% increased risk of MACE (adjusted HR = 1.858, 95% CI 1.038–3.326, *P* = 0.037), indicating a stepwise increase in MACE risk with decreasing QFR. In the sensitivity analysis, when treatment was excluded from the model, the adjusted HR for QFR group was 1.898 (95% CI: 1.078–3.341, *P* = 0.026), with a change of less than 5%, supporting the robustness of the results. Multivariable Firth penalized likelihood Cox regression was also performed to validate the robustness of the results (adjusted HR = 1.852, 95% CI 1.038–3.312, *P* = 0.037, Supplementary Table S3).

**Table 4:** Multivariable Cox regression model for MACE.

Variable (Ref)	HR	95% CI		*P* value
QFR Group (High QFR)	1.858	1.038	3.326	0.037
SYNTAX Score (Low Risk)	1.855	1.181	2.914	0.007
eGFR (≥60 mL/min/1.73m^2^)	3.078	1.067	8.877	0.037
Treatment (Medication)	1.158	0.530	2.528	0.713

QFR Group as an ordinal variable.

Pairwise comparisons of QFR categories were performed in the multivariable model. Patients in the Low QFR group had the highest risk compared with the High QFR group (adjusted HR = 3.296, 95% CI 1.098–9.891, *P* = 0.033), while the Grey-Zone QFR group showed intermediate risk (Grey-Zone vs. High HR = 1.395, 95% CI 0.524–3.714, *P* = 0.505; Low vs. Grey-Zone HR = 2.362, 95% CI 0.977–5.710, *P* = 0.056, Supplementary Table S4).

We further evaluated vessel-specific outcomes, focusing on target vessel myocardial infarction (TV-MI) and ischemia-driven target vessel revascularization (ID-TVR). During follow-up, 13 patients experienced ID-TVR, including 4 patients with TV-MI. Univariable Firth penalized Cox regression analyses demonstrated that QFR as a continuous variable was inversely associated with ID-TVR (HR = 0.896 per 0.01-unit increase, 95% CI 0.821–0.975, *P* = 0.011), and QFR Group (from High to Low Group) was significant (HR = 2.391, 95% CI 1.092–5.381, *P* = 0.029, Supplementary Table S5). In multivariable Firth Cox regression adjusting for SYNTAX score and treatment, QFR Group remained an independent predictor of ID-TVR (adjusted HR = 2.333, 95% CI 1.004–5.510, *P* = 0.049, Supplementary Table S6).

**Figure 3:**
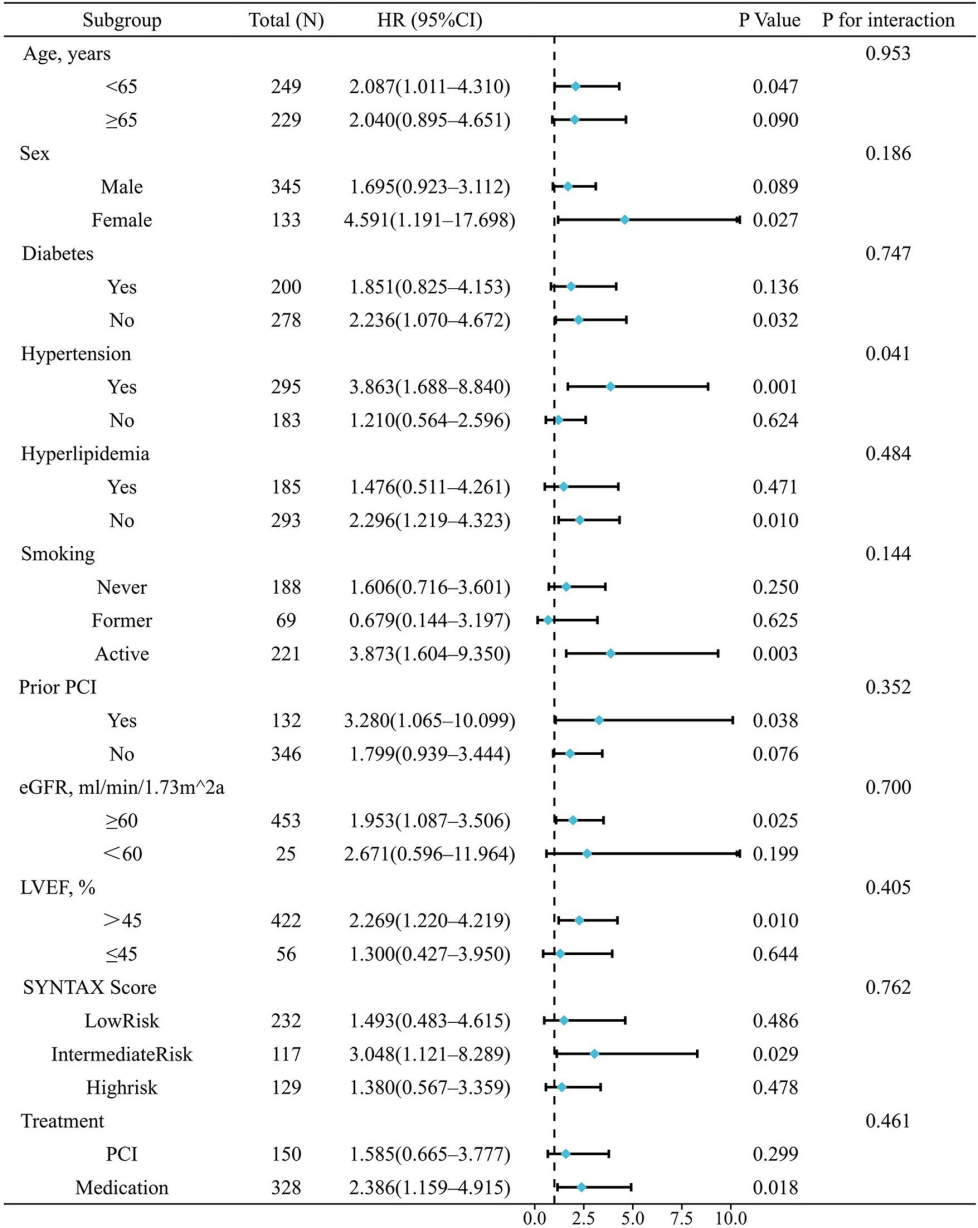
Subgroup and interaction analyses for the association between QFR group and MACE.

To explore whether the predictive value of QFR varies across clinical subgroups, we performed subgroup analyses and interaction analyses based on Cox regression. Subgroups included age, sex, diabetes, hypertension, hyperlipidemia, smoking status, prior PCI, eGFR, LVEF, SYNTAX risk group, and treatment strategy. As shown in Figure 3, no significant interactions were observed in any subgroup except for hypertension. In hypertensive patients, QFR was strongly predictive of MACE (HR = 3.863, 95% CI: 1.688–8.840, *P* = 0.001), whereas in non-hypertensive patients, QFR was not significantly associated with MACE (HR = 1.210, 95% CI: 0.564–2.596, *P* = 0.624; *P* for interaction = 0.041).

Some subgroups had few events, which led to wide confidence intervals. Therefore, these results should be interpreted with caution. Sensitivity analyses using Firth penalized likelihood Cox showed trends consistent with the main findings (Supplementary Table S7).

## 4 Discussion

In this retrospective cohort analysis based on a real-world clinical population guided by conventional angiography, we analysed patients with CAD who were identified as having single-vessel intermediate stenosis. The main finding was a continuous, stepwise increase in the risk of adverse events across the QFR spectrum of 0.70–0.90, particularly from High through the grey zone to Low QFR groups, highlighting its prognostic value.

As a novel physiological tool, quantitative flow ratio (QFR) enables FFR simulation based on angiographic images without pressure wires or hyperemic agents. However, there is still insufficient evidence regarding the predictive value of baseline QFR for prognosis and risk stratification, especially for grey zone and adjacent segments.

Choi et al. (16) and Buono et al. (17) have demonstrated that baseline QFR≤0.80 is associated with worse outcomes, but these analyses used a single dichotomous threshold 0.80 across a wide range and did not evaluate the grey zone specifically. As a result, the prognostic differences they observed may have been influenced by extreme QFR values. Similar to the continuous risk observed across the FFR grey zone (0.75–0.80) and its adjacent segments as reported by Adjedj et al. (18), QFR values within and around the grey zone may also represent a transitional risk spectrum. This retrospective study is the first to evaluate the prognostic value of baseline QFR by focusing specifically on the grey zone and adjacent segments. Our findings demonstrate a continuous increase in MACE risk across the QFR range of 0.70–0.90, with progressively higher risk at lower QFR values. This excess risk was mainly driven by MI and IDR. Although the overall incidence of TVF did not reach statistical significance, a clear increasing trend was observed, driven primarily by significant differences in ID-TVR. These results support the role of QFR in predicting vessel-level adverse events. Consistently, Cox regression confirmed that QFR was independently associated with MACE and ID-TVR, showing predictive value both as a continuous and ordinal variable. Our observations echo the findings of Adjedj et al. (18), demonstrating that QFR, like FFR, exhibits a continuous risk gradient rather than a binary pattern in the grey zone and adjacent ranges.

Notably, due to the limited number of events, the confidence interval for the QFR group's HR in the multivariable Cox model was wide. Although Firth penalization was applied to reduce small-sample bias and the multivariable Cox model was used to adjust for potential confounders, these estimates should still be interpreted cautiously given the retrospective nature of the study. Additionally, the study was underpowered to detect associations with low-frequency outcomes such as cardiac death or all-cause mortality; larger, prospective studies with longer follow-up are needed to assess these endpoints.

Exploratory subgroup and interaction analyses showed that the association between QFR and MACE was generally consistent across most clinical subgroups, with no significant effect modification. Hypertension was the only subgroup with a statistically significant interaction (*P* for interaction = 0.041). QFR was prognostic in hypertensive but not in non-hypertensive patients. Several exploratory explanations may account for this observation. Firstly, we found that patients with hypertension had lower rates of non-target-vessel events (including cardiac death, non-target-vessel MI, and non-target-vessel IDR, adjusted HR = 0.287, 95% CI: 0.097–0.797, *P* = 0.017), which are not directly related to the QFR-assessed vessel. This may have made MACE in hypertensive patients more strongly driven by target-vessel events, thereby amplifying the apparent prognostic contribution of QFR. Secondly, prior studies report that discordance between resting Pd/Pa and FFR is more common in non-hypertensive patients (19), which may reflect greater variability in resting coronary hemodynamic, and reduce the predictive accuracy of angiography-derived physiological indices like QFR (19). These interpretations remain speculative and hypothesis-generating. Given the limited sample size, low event numbers in several subgroups, and the exploratory nature of these analyses, the findings should be interpreted with caution and warrant confirmation in larger prospective studies.

Stratified Kaplan–Meier analyses by treatment strategy showed that QFR retained prognostic significance in patients managed with medication, whereas no significant association was observed in the PCI group, consistent with the subgroup analysis results. This pattern aligns with prior FFR-based observations reported by Adjedj et al. (18), suggesting that once a vessel is revascularized, the prognostic value of its baseline physiological severity diminishes. In patients with low QFR, PCI likely improves hemodynamics and thereby attenuates baseline QFR's predictive power, while in high-QFR lesions, PCI may introduce additional risk. Although, the interaction between QFR and treatment strategy was not statistically significant in the Cox regression model, a non-significant interaction should not be interpreted as evidence of equivalence. Given the modest sample size and low event rates, our study was likely underpowered to formally test this interaction, despite the overall trend—where QFR predicted events primarily in medically managed patients—being directionally consistent with previous FFR-based studies (18) and physiologically plausible. These findings underscore the need for larger prospective studies to clarify the interplay between QFR and treatment strategy in determining long-term outcomes.

### 4.1 Limitations

This study has several limitations. First, as a single-center retrospective study, inherent selection and treatment biases are unavoidable. Treatment decisions were influenced by lesion severity, which correlates with baseline QFR. Although multivariable Cox regression adjusted for key covariates such as SYNTAX score and treatment strategy, residual confounding from unmeasured factors, including plaque characteristics, microvascular dysfunction, operator preference, and patient factors, cannot be excluded, and causal inference cannot be made. Second, the total number of events was small, resulting in wide confidence intervals for hazard ratios and limited statistical power. Despite using Firth penalization to validate the robustness of the results and reduce bias, these findings should still be interpreted cautiously. Third, while MACE was used as the primary endpoint to reflect overall coronary disease, it includes both target- and non-target-vessel events, which may dilute the specificity of the QFR-outcome association; in contrast, analyses of target vessel IDR (ID-TVR) offered greater specificity, but the study remained underpowered for some individual components. Finally, as only single-vessel intermediate stenosis was included, caution is warranted when extrapolating these findings to patients with complex multivessel disease. Larger prospective and randomized studies with longer follow-up are needed to validate these findings.

## 5 Conclusion

Baseline QFR and its three-tier stratification across the grey-zone and adjacent ranges show a continuous association with adverse events in single-vessel intermediate coronary stenosis, supporting its role in functional evaluation, risk stratification, and prognostic prediction.

## Data Availability

The raw data supporting the conclusions of this article will be made available by the authors, without undue reservation.
