# Supplementary material for: Prognostic value of baseline QFR in single-vessel intermediate coronary stenosis
Source: Front Cardiovasc Med. 2026 Jan 9;12:1726729. doi: 10.3389/fcvm.2025.1726729 (PMC12827562; doi:10.3389/fcvm.2025.1726729)
Supplement: Supplementary file 1 [file Datasheet1.docx]

**Supplementary material:**

**Table S1**

Table S1 presents the log-rank test P values adjusted for treatment stratification, as well as the log-rank test P values for the association between QFR grouping and clinical outcomes within each treatment stratum.

Table S1. The P values in Kaplan–Meier Analysis of Clinical Outcomes by QFR Groups Stratified by Treatment Strategy

| **Clinical Outcomes** | **Total** | | **PCI** | **Medication** |
| --- | --- | --- | --- | --- |
|  | Unadjusted P value | Adjusted P value | P value | P value |
| **MACE** | 0.008 | 0.030 | 0.510 | 0.028 |
| **Cardiac death** | 0.569 | 0.599 | 0.915 | 0.340 |
| **MI** | 0.029 | 0.105 | 0.036 | 0.205 |
| **IDR** | 0.004 | 0.019 | 0.525 | 0.012 |
| **TVF** | 0.051 | 0.103 | 0.645 | 0.030 |
| **Cardiac death** | 0.569 | 0.599 | 0.915 | 0.340 |
| **TV-MI** | 0.136 | 0.254 | 0.193 | 0.205 |
| **ID-TVR** | 0.027 | 0.023 | 0.994 | 0.008 |

**Table S2**

Table S2 Univariable Cox regression for MACE

| **Variable (Ref)** | **HR** | **95% CI** | | **P value** |
| --- | --- | --- | --- | --- |
| **Age (<65)** | 0.782 | 0.370 | 1.653 | 0.519 |
| **Sex (Female)** | 1.770 | 0.673 | 4.657 | 0.247 |
| **BMI (<30 kg/m^2^)** | 0.567 | 0.077 | 4.172 | 0.577 |
| **Diabetes mellitus (No)** | 1.219 | 0.580 | 2.561 | 0.602 |
| **Hypertension (No)** | 0.612 | 0.292 | 1.283 | 0.193 |
| **Hyperlipidemia (No)** | 0.754 | 0.341 | 1.666 | 0.485 |
| **Smoking (No)** | 0.915 | 0.613 | 1.365 | 0.663 |
| **Prior PCI (No)** | 0.864 | 0.367 | 2.032 | 0.737 |
| **Prior MI (No)** | 0.791 | 0.275 | 2.281 | 0.665 |
| **RBC** | 0.509 | 0.277 | 0.937 | 0.083 |
| **WBC** | 0.929 | 0.767 | 1.126 | 0.452 |
| **PLT** | 1.000 | 0.993 | 1.006 | 0.906 |
| **ALB** | 0.907 | 0.840 | 0.979 | 0.013 |
| **FIB** | 1.313 | 0.960 | 1.796 | 0.089 |
| **FDP** | 0.985 | 0.852 | 1.139 | 0.837 |
| **LDL-C** | 1.149 | 0.755 | 1.748 | 0.517 |
| **HbA1c** | 1.038 | 0.786 | 1.372 | 0.791 |
| **eGFR Group (≥60 ml/min/1.73m^2^)** | 3.266 | 1.132 | 9.417 | 0.029 |
| **LVEF (>45%)** | 2.224 | 0.902 | 5.486 | 0.083 |
| **QFR (per 0.01unit)** | 0.928 | 0.875 | 0.984 | 0.012 |
| **QFR Group (High QFR)** | 2.067 | 1.200 | 3.558 | 0.009 |
| **SYNTAX Group (Low Risk)** | 1.967 | 1.260 | 3.068 | 0.003 |
| **Treatment (Medication)** | 1.423 | 0.667 | 3.039 | 0.362 |
| QFR Group as an ordinal variable. | | | | |

**Table S3**

A Firth penalized likelihood Cox regression was performed as a sensitivity analysis. The results were consistent with the primary multivariable model.

Table S3 Multivariable Firth penalized likelihood Cox regression for MACE

| **Variable (Ref)** | **HR** | **95% CI** | | **P value** |
| --- | --- | --- | --- | --- |
| **QFR Group (High QFR)** | 1.852 | 1.038 | 3.312 | 0.037 |
| **SYNTAX Score (Low Risk)** | 1.832 | 1.185 | 2.904 | 0.006 |
| **eGFR (≥60 ml/min/1.73m^2^)** | 3.378 | 1.071 | 8.407 | 0.039 |
| **Treatment (Medication)** | 1.177 | 0.532 | 2.509 | 0.679 |

QFR Group as an ordinal variable.

**Table S4**

Table S4 Pairwise comparisons of QFR groups in multivariable Cox proportional hazards models treating QFR as a categorical variable, adjusted for eGFR, SYNTAX score, and treatment strategy.

| **Comparison (Ref)** | **HR** | **95% CI** | | **P value** |
| --- | --- | --- | --- | --- |
| **Low vs. High QFR (High)** | 3.296 | 1.098 | 9.891 | 0.033 |
| **Grey-zone vs. High QFR (High)** | 1.395 | 0.524 | 3.714 | 0.505 |
| **Low vs. Grey-Zone QFR (Grey-Zone)** | 2.362 | 0.977 | 5.710 | 0.056 |

**Table S5**

Table S5 Univariable Firth penalized likelihood Cox regression for ID-TVR

| **Variable (Ref)** | **HR** | **95% CI** | | **P value** |
| --- | --- | --- | --- | --- |
| **Age (<65)** | 0.498 | 0.146 | 1.460 | 0.208 |
| **Sex (Female)** | 1.758 | 0.519 | 9.048 | 0.392 |
| **BMI (<30 kg/m^2^)** | 0.564 | 0.004 | 4.242 | 0.663 |
| **Diabetes mellitus (No)** | 1.220 | 0.411 | 3.526 | 0.712 |
| **Hypertension (No)** | 0.948 | 0.330 | 2.968 | 0.923 |
| **Hyperlipidemia (No)** | 0.531 | 0.134 | 1.634 | 0.283 |
| **Smoking (No)** | 0.991 | 0.558 | 1.786 | 0.976 |
| **Prior PCI (No)** | 0.552 | 0.107 | 1.870 | 0.366 |
| **Prior MI (No)** | 1.587 | 0.401 | 4.882 | 0.473 |
| **RBC** | 0.588 | 0.247 | 1.544 | 0.273 |
| **PLT** | 0.998 | 0.988 | 1.007 | 0.703 |
| **ALB** | 0.972 | 0.863 | 1.094 | 0.634 |
| **FIB** | 1.427 | 0.935 | 2.178 | 0.099 |
| **FDP** | 0.890 | 0.572 | 1.384 | 0.605 |
| **LDL-C** | 1.307 | 0.735 | 2.323 | 0.362 |
| **HbA1c** | 1.130 | 0.789 | 1.617 | 0.506 |
| **eGFR Group (≥60 ml/min/1.73m^2^)** | 0.688 | 0.005 | 5.173 | 0.783 |
| **LVEF (>45%)** | 1.721 | 0.334 | 5.830 | 0.463 |
| **QFR (per 0.01unit)** | 0.896 | 0.821 | 0.975 | 0.011 |
| **QFR Group (High QFR)** | 2.391 | 1.092 | 5.381 | 0.029 |
| **SYNTAX Group (Low Risk)** | 2.068 | 1.110 | 4.121 | 0.022 |
| **Treatment (Medication)** | 1.034 | 0.302 | 3.030 | 0.954 |

**Table S6**

Table S6 Multivariable Firth penalized likelihood Cox regression for ID-TVR

| **Model** | **Variable (Ref)** | **HR** | **95% CI** | | **P value** |
| --- | --- | --- | --- | --- | --- |
| **Model 1** | **QFR Group (High QFR)** | 2.566 | 1.134 | 5.983 | 0.024 |
|  | **Treatment (Medication)** | 0.728 | 0.205 | 2.237 | 0.588 |
| **Model 2** | **QFR Group (High QFR)** | 2.333 | 1.004 | 5.510 | 0.049 |
|  | **Treatment (Medication)** | 0.787 | 0.225 | 2.372 | 0.679 |
|  | **SYNTAX Score (Low Risk)** | 1.903 | 1.010 | 3.828 | 0.046 |

QFR Group as an ordinal variable.

**Table S7**

Subgroup analyses and interaction analyses were performed using Firth penalized likelihood Cox regression, the results showed the trends were consistent with the main findings.

Table S7 Subgroup analyses and interaction analyses were performed using Firth penalized likelihood Cox regression for MACE.

| **Variable (Ref)** | **HR** | **95% CI** | | **P value** | **P for interaction** |
| --- | --- | --- | --- | --- | --- |
| Age, years |  |  |  |  | 0.949 |
| <65 | 2.080 | 1.015 | 4.298 | 0.045 |  |
| ≥65 | 2.027 | 0.902 | 4.631 | 0.087 |  |
| Sex |  |  |  |  | 0.068 |
| Male | 1.694 | 0.925 | 3.106 | 0.088 |  |
| Female | 4.215 | 1.259 | 17.301 | 0.019 |  |
| Diabetes |  |  |  |  | 0.741 |
| Yes | 1.841 | 0.829 | 4.138 | 0.133 |  |
| No | 2.227 | 1.076 | 4.658 | 0.031 |  |
| Hypertension |  |  |  |  | 0.040 |
| Yes | 3.767 | 1.709 | 8.833 | <0.001 |  |
| No | 1.228 | 0.566 | 2.572 | 0.593 |  |
| Hyperlipidemia |  |  |  |  | 0.493 |
| Yes | 1.485 | 0.515 | 4.233 | 0.460 |  |
| No | 2.276 | 1.226 | 4.317 | 0.009 |  |
| Smoking |  |  |  |  | 0.146 |
| Never | 1.617 | 0.720 | 3.573 | 0.239 |  |
| Former | 0.712 | 0.149 | 3.080 | 0.651 |  |
| Active | 3.768 | 1.627 | 9.334 | 0.002 |  |
| Prior PCI |  |  |  |  | 0.384 |
| Yes | 3.052 | 1.112 | 9.988 | 0.048 |  |
| No | 1.802 | 0.941 | 3.436 | 0.075 |  |
| eGFR, ml/min/1.73m^2a |  |  |  |  | 0.734 |
| ≥60 | 1.950 | 1.090 | 3.501 | 0.025 |  |
| ＜60 | 2.529 | 0.623 | 11.631 | 0.193 |  |
| LVEF, % |  |  |  |  | 0.393 |
| ＞45 | 2.261 | 1.224 | 4.214 | 0.009 |  |
| ≤45 | 1.299 | 0.435 | 3.880 | 0.644 |  |
| SYNTAX Score |  |  |  |  | 0.767 |
| Low Risk | 1.534 | 0.490 | 4.497 | 0.444 |  |
| Intermediate Risk | 2.936 | 1.146 | 8.251 | 0.025 |  |
| High risk | 1.380 | 0.568 | 3.350 | 0.476 |  |
| Treatment |  |  |  |  | 0.439 |
| PCI | 1.560 | 0.672 | 3.763 | 0.301 |  |
| Medication | 2.386 | 1.162 | 4.901 | 0.018 |  |

QFR Group as an ordinal variable.

**Figure S1**

Restricted cubic spline (RCS) analysis demonstrated an inverse association between QFR and the risk of MACE. As QFR decreased, the hazard ratio for MACE increased progressively. The overall association was statistically significant (P=0.026). The test for non-linearity was not significant (P for nonlinear=0.538), suggesting that the relationship between QFR and MACE risk was approximately linear.


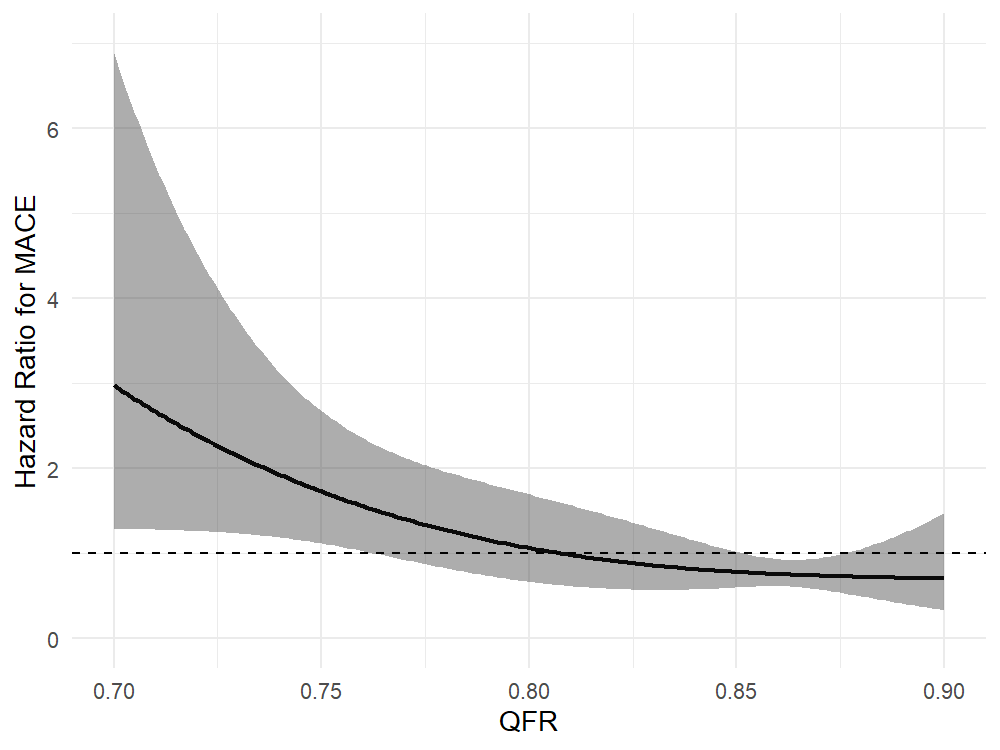


Figure S1 Restricted Cubic Spline (RCS) Curve for the Association Between QFR and Risk of MACE. (knots=3, P=0.026, P for nonlinear=0.538)
